# The Association Between Long Working Hours of Parents and Dyslipidemia in Their Children

**DOI:** 10.3389/fpubh.2022.894609

**Published:** 2022-06-30

**Authors:** Joonho Ahn, Dong-Wook Lee, Mo-Yeol Kang, Jun-Pyo Myong, Mi Hae Chung, Hyoung-Ryoul Kim, Jongin Lee

**Affiliations:** ^1^Department of Occupational and Environmental Medicine, Seoul St. Mary's Hospital, College of Medicine, The Catholic University of Korea, Seoul, South Korea; ^2^Department of Preventive Medicine, College of Medicine, Seoul National University, Seoul, South Korea; ^3^Department of Pediatrics, Seoul St. Mary's Hospital, College of Medicine, The Catholic University of Korea, Seoul, South Korea

**Keywords:** mothers, work schedule tolerance, child, dyslipidemia, cholesterol, LDL

## Abstract

The purpose of our study is to examine the association between children's low-density lipoprotein cholesterol (LDL-C) levels and their parents' working hours. We used data from the 2010–2018 Korea National Health and Nutrition Examination Survey in which lipid profile samples of 3,799 children were eligible. Logistic regression analyses were used with an outcome variable of the dichotomous LDL-C group and an exposure variable of the father's and mother's working hours, respectively. In logistic regression models adjusted for age, sex, household income, education level of children and parents, and working hours of the parents, mothers' working hours more than 52 h per week were significantly associated with their children's dyslipidemia [OR 2.14, 95% confidence interval (CI) 1.33–3.47] compared to working 40 h or less, whereas fathers' working hours did not show statistical significance (OR 1.08, 95% CI 0.71–1.66) in the same manner. The association was greatest for elementary school students for mothers working more than 52 h per week (OR 3.42, 95% CI 1.64–7.14) compared to those working hours 40 h per week or less. Mothers' long working hours were associated with a higher prevalence of dyslipidemia in their children. The association was strongest for elementary school students. Proper working time of parents is required for their children's health.

## Introduction

Working for long hours is a well-known factor with a negative influence on cardiovascular health. Several previous studies have suggested that the risks for cerebro-cardiovascular diseases (CVDs), such as coronary heart disease or stroke, are increased by long working hours (1). The risk factors for CVDs can be estimated by age, sex, diabetes mellitus, blood pressure, smoking status, and cholesterol levels (2). Risk elevation of CVDs by working for long hours is believed to be associated with these risk factors. For example, long working hours are an independent risk factor for hypertension (3). It is also associated with prediabetic status or poor glycemic control in patients diagnosed with diabetes mellitus (4, 5).

Low-density lipoprotein cholesterol (LDL-C) is also a risk factor for CVDs *via* atherosclerosis (6). Lower level of LDL-C can reduce the risk of CVDs (7). The association between working time and LDL-C has also been examined in some studies; truck drivers with irregular shifts showed higher LDL-C levels than that of day-shift workers (8). Another study involving truck drivers in the United States showed that daily work hours were associated with LDL-C (9).

On the other hand, a worker's working hours may affect his/her family's health. A study by Kang and Hong is an example, which showed that the risks for CVDs in the future were associated with spouses' long working hours (10). This observation can be explained by health behavior, especially behaviors related to food consumption because of the family's long working hours. For example, women with husbands of long working hours were associated with predicted fast-food consumption, even though the association was not linear (11).

In this context, it is probable that the working hours of parents can have an effect on their children's food habits. The most important factor that surrounds children's eating behavior is parental food habits (12). Adequate nutrition is more important for children and adolescents than for adults. Food consumption behaviors can be related to the lipid profile of children (13), and prevention and management of atherosclerosis and CVD risk factors should begin early in life (14). However, there has been no study on the effect of long working hours of parents on the risk of CVD in their children.

LDL-C is considered an appropriate target because it causes atherosclerotic CVD (15), and lipid-lowering therapy is an important strategy for primary and secondary prevention of CVD (16, 17). Over the past few decades, LDL-C has been recommended as the primary therapeutic goal for lipid management in patients with CVD (18). The risk of LDL-C is also well established in children and adolescents (19, 20). Therefore, we tried to reveal the association of parents' working time with their children's health. The aim of this study was to examine the association between children's LDL-C levels and their parents' working hours.

## Subjects and Methods

### Study Participants

We used data from the Korea National Health and Nutrition Examination Survey (KNHANES) (21). The KNHANES is an annual cross-sectional survey conducted by the Korea Centers for Disease Control and Prevention, which represents the entire population of the Republic of Korea. The KNHANES sampling plan followed a multi-stage clustered probability design, and each wave of the survey was conducted for 3 years. The latest wave was from 2016 to 2018 (seventh wave). The main framework of the survey was the same for each year; however, some additional variables were added or deleted by each wave or survey year. We analyzed data from the fifth to seventh waves, that is, nine survey years from 2010 to 2018. Approximately 8,000 people recruited with structured sampling participated in the survey. The total number of participants was 72,501. We included participants from two-generation families; those from other forms of families, such as couples, three-generation families, or one-person households, were excluded. As a result, 35,882 participants were finally included. Of these, 11,306 were children (age <19 years).

**Figure 1:**
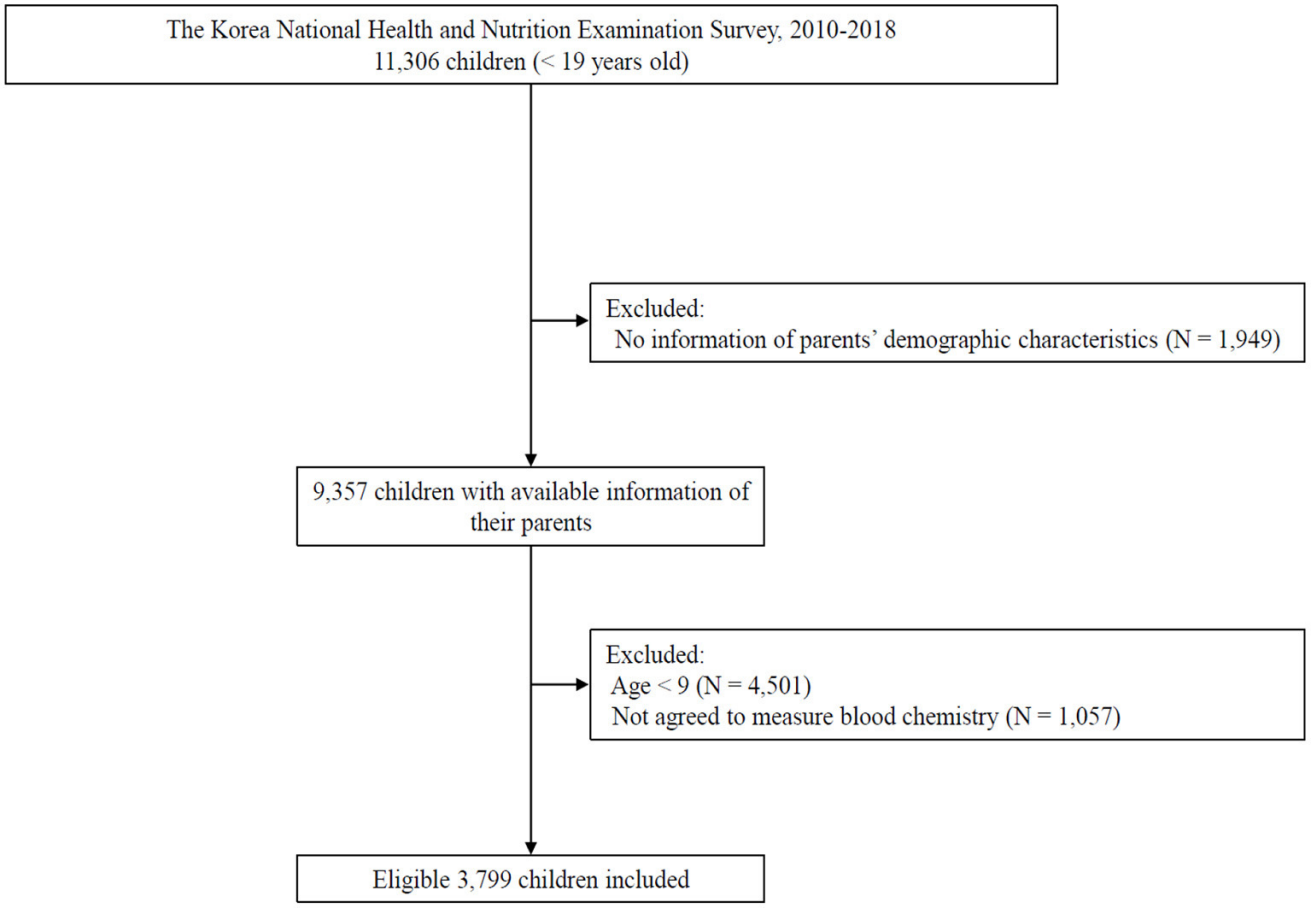
Flow diagram of defining study subjects.

Because KNHANES is based on systematic sampling of target households for primary sampling units, most of the young participants were surveyed with their parents simultaneously. To explore the difference in working hours between fathers and mothers and reduce potential bias from inaccurate answers to questionnaire items, we excluded children whose parents' information on working hours was not included in the KNHANES. Hence, 9,357 children with information from both parents were included in the study. Blood chemistry battery profile of the study participants aged ≥9 years was measured in KNHANES after obtaining informed consent. We excluded children whose lipid profiles were not measured. Finally, 3,799 children were included in the study (Figure 1).

### Working Hours of Parents

Questionnaire items dealing with working conditions, including working hours, are assessed for all participants of KNHANES every year. They are required to respond to the following open-ended question: “What are the average hours at work per week, including overtime or night work? Mealtime is excluded.” Because the Korean Labor Standards Act limits maximum working hours per week to 40 h, we defined participants with working hours ≤40 h, as a reference group. Participants who did not work were also included in this group. The Labor Standard Act also limits overtime work to 52 h per week. Therefore, we divided the participants into 2 groups based on the following working hours: 41–52 h per week and >52 h per week. The distribution of working hours for fathers and mothers is described in Supplementary Table 1.

### Demographic Characteristics of Children

We used basic information on sex, age, education level, and household income from the KNHANES. Because the eligible participant children were 9 years old or above, we divided the participants into the following two groups based on their age: 9–13 years (children) and 13–19 years (adolescent). Based on the standard quartile income value, household income level is assessed every year and classified as follows: 1st quartile (low income), 2nd quartile (middle-low income), 3rd quartile (middle-high income), and 4th quartile (high income). The educational level consists of 3 levels as follows: elementary school, middle school, and high school or above. The educational level of parents of the children is also considered.

### Low-Density Lipoprotein Cholesterol

The blood chemistry battery examination of KNHANES includes total cholesterol (TC), triglyceride (TG), and high-density lipoprotein cholesterol (HDL-C). We assessed the LDL-C levels of all the data available using Friedwald's method (22). Measurement of TC, TG, and HDL-C was performed with a Hitachi Automatic Analyzer 7600-210 (Hitachi, Japan) using the enzymatic method (TC and TG) and homogeneous enzymatic colorimetric method (HDL-C). The reagents used were Pureauto SCHO-N (Sekisui/JAPAN) for the enzymatic method and Cholestest N HDL (Sekisui/JAPAN) for the homogeneous enzymatic colorimetric method. KNHANES has been participating in the Lipid Standardization Program run by the United States Centers for Disease Control and Prevention since 2009. Because the level of LDL-C ≥ 130 mg/dl is considered “high LDL-C” or pediatric dyslipidemia (23), we set the same criteria to define the high-LDL children group. There was no child on treatment for dyslipidemia in this survey.

### Statistical Analysis

The association between the demographic characteristics of the study participants and their level of LDL-C was examined using chi-square tests. The characteristics were also examined with weekly working hours of the father and mother, respectively, with chi-square tests for discrete variables and *t*-test for continuous variables (concentration of LDL-C).

**Figure 2:**
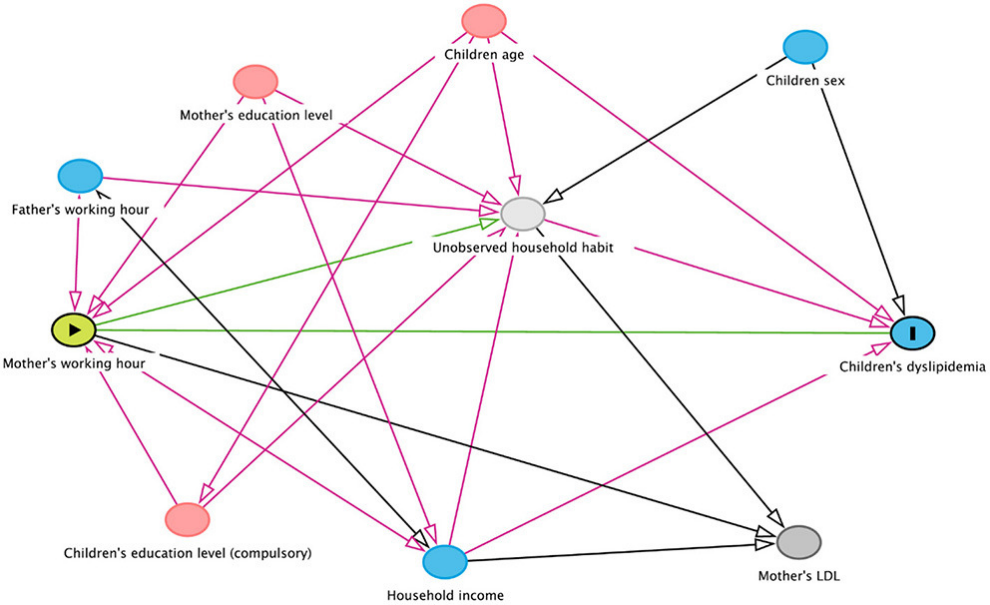
A diagram showing the possible association between mother's working hours and children's dyslipidemia.

Logistic regression analyses were performed with an independent variable of the dichotomous LDL-C group and a dependent variable of the father's and mother's working hours, respectively. We also examined an adjusted model. In order to select appropriate variables for adjustment, we established a diagram of directed acyclic graph (DAG). Assuming an association between mother's working hour and children's dyslipidemia *via* unobserved household habits, appropriate adjustment variables were children's age, children's education level, father's working hour, household income, mother's education level (Figure 2). The same assumption was applied to in case of focusing the relationship between father's working hour and children's dyslipidemia. Stratified models with education level were also established to examine the effects of different school levels. Nightshift, as well as long working time, is considered to influence dyslipidemia of the workers' children. Therefore, an additional adjusted model with rotating shift type of fathers and mothers respectively were also established.

All statistical analyses were performed with R program version 4.1.1 (Vienna, Austria) with the package “survey” to calculate with the survey-weights of KNHANES. Statistical significance was set at *P* < 0.05 or 95% confidence interval (CI) not containing 1.00.

## Results

Childhood dyslipidemia, defined as an LDL-C level of 130 mg/dL or above, was significantly associated with demographic characteristics, such as age, sex, household income, and education level of children and their mothers. In the low-LDL-C group, the proportion of adolescents (age ≥ 13 and <19 years, 64.8%) was relatively higher than in the dyslipidemia group. Males were dominant in the low-LDL-C group (53.5%), while females were dominant in the dyslipidemia group (56.2%). Both groups showed high distribution in the high-income groups, including the 3rd and 4th quartiles. However, a higher proportion of those with the highest income was found in the dyslipidemia group (43.7%). The education level of middle school was the highest in the low LDL-C group (35.5%), while that of elementary school was dominant (38.8%) in the dyslipidemia group (Table 1).

**Table 1:** Baseline characteristics of study participants divided by low-density lipoprotein cholesterol (LDL-C) levels.

	**Level of LDL-C**				
	**<130 mg/dl**		**≥130 mg/dl**		***P*** value^*^
	**(*****N*** **= 3,566)**		**(*****N*** **= 233)**		
**Age (years)**					0.025
≥ 9 and <13	1,254	(35.2%)	99	(42.5%)	
≥ 13 and <19	2,312	(64.8%)	134	(57.5%)	
**Sex**					0.005
Male	1,907	(53.5%)	102	(43.8%)	
Female	1,659	(46.5%)	131	(56.2%)	
**Household income**					0.002
1st quartile	161	(4.5%)	14	(6.1%)	
2nd quartile	815	(22.9%)	35	(15.2%)	
3rd quartile	1,290	(36.3%)	81	(35.1%)	
4th quartile	1,287	(36.2%)	101	(43.7%)	
**Education level—children**					0.011
Elementary school	1,102	(31.1%)	90	(38.8%)	
Middle school	1,259	(35.5%)	57	(24.6%)	
Above high school	1,187	(33.5%)	85	(36.6%)	
**Education level—father**					0.262
Middle school	184	(5.2%)	17	(7.3%)	
High school	1,191	(33.6%)	76	(32.8%)	
Above college	1,692	(47.7%)	119	(51.3%)	
**Education level—mother**					0.011
Middle school	110	(3.1%)	9	(3.9%)	
High school	1,719	(48.4%)	109	(47.0%)	
Above college	1,511	(42.6%)	99	(42.7%)	

* *Chi-square test*.

Regarding fathers' working hours, the demographic characteristics did not show differences among the 3 groups (working hours ≤40 h, 41–52 h, and >52 h per week), except for household income and educational level of fathers. The distribution of high income was higher in the ≤40 h group, while that of low income was higher in the >52 h group (*P* < 0.001). LDL-C levels were not significantly correlated with fathers' working hours (Table 2).

**Table 2:** Baseline characteristics of study participants divided by working hours of the father.

	**Father's weekly working hours**					
	**≤40**		**>40 and ≤52**		**>52**		***P*** value^*^
	**(*****N*** **= 1,633)**		**(*****N*** **= 1,130)**		**(*****N*** **= 1,036)**		
**Age (years)**							0.350
≥9 and <13	571	(35.0%)	428	(37.9%)	354	(34.2%)	
≥13 and <19	1,062	(65.0%)	702	(62.1%)	682	(65.8%)	
**Sex**							0.316
Male	880	(53.9%)	571	(50.5%)	558	(53.9%)	
Female	753	(46.1%)	559	(49.5%)	478	(46.1%)	
**Household income**							<0.001
1st quartile	96	(5.9%)	31	(2.7%)	48	(4.7%)	
2nd quartile	311	(19.1%)	238	(21.1%)	301	(29.3%)	
3rd quartile	542	(33.3%)	438	(38.8%)	391	(38.0%)	
4th quartile	678	(41.7%)	421	(37.3%)	289	(28.1%)	
**Education level—children**						0.228
Elementary school	511	(31.5%)	377	(33.5%)	304	(29.4%)	
Middle school	556	(34.3%)	401	(35.6%)	359	(34.8%)	
Above high school	553	(34.1%)	349	(31.0%)	370	(35.8%)	
**Education level—father**							<0.001
Middle school	57	(3.5%)	47	(4.2%)	97	(9.4%)	
High school	378	(23.3%)	446	(39.6%)	443	(42.9%)	
Above college	723	(44.6%)	619	(54.9%)	469	(45.4%)	
**Low-density lipoprotein**							0.376
<130 mg/dl	1,544	(94.5%)	1,056	(93.5%)	966	(93.2%)	
≥ 130 mg/dl	89	(5.5%)	74	(6.5%)	70	(6.8%)	

* *Chi-square test*.

The working hours of the mothers showed different patterns. Higher age and educational level of the children were significantly associated with mothers' working hours. The prevalence of dyslipidemia was significantly higher in the group with mothers working for more than 52 h per week (9.0%, *P* = 0.017, Table 3).

**Table 3:** Baseline characteristics of study participants divided by working hours of the mother.

	**Mother's weekly working hour**						
	**≤40**		**>40 and ≤52**		**>52**		
	**(*****N*** **= 2,937)**		**(*****N*** **= 516)**		**(*****N*** **= 346)**		***P*** value^*^
**Age (years)**							0.001
≥9 and <13	1,101	(37.5%)	156	(30.2%)	96	(27.7%)	
≥13 and <19	1,836	(62.5%)	360	(69.8%)	250	(72.3%)	
**Sex**							0.552
Male	1,572	(53.5%)	261	(50.6%)	176	(50.9%)	
Female	1,365	(46.5%)	255	(49.4%)	170	(49.1%)	
**Household income**							0.084
1st quartile	143	(4.9%)	18	(3.5%)	14	(4.1%)	
2nd quartile	641	(21.9%)	110	(21.3%)	99	(28.8%)	
3rd quartile	1,028	(35.2%)	208	(40.3%)	135	(39.2%)	
4th quartile	1,112	(38.0%)	180	(34.9%)	96	(27.9%)	
**Education level—children**						<0.001
Elementary school	972	(33.3%)	140	(27.1%)	80	(23.1%)	
Middle school	1,016	(34.8%)	185	(35.9%)	115	(33.2%)	
Above high school	930	(31.9%)	191	(37.0%)	151	(43.6%)	
**Education level—mother**						<0.001
Middle school	75	(2.6%)	22	(4.3%)	22	(6.4%)	
High school	1,332	(45.6%)	261	(50.6%)	235	(67.9%)	
Above college	1,313	(45.0%)	220	(42.6%)	77	(22.3%)	
**Low-density lipoprotein**							0.017
<130 mg/dL	2,774	(94.5%)	477	(92.4%)	315	(91.0%)	
≥130 mg/dL	163	(5.5%)	39	(7.6%)	31	(9.0%)	

* *Chi-square test*.

In crude models of logistic regression analyses with the independent variable of dyslipidemia, fathers' working hours showed higher odds ratios (ORs), but they were not statistically significant at 95% CIs. However, mothers working more than 52 h per week (OR 1.81, 95% CI 1.14–2.88) showed higher ORs than those working 40 h or less. The association showed greater ORs in the adjusted model (OR 2.14, 95% CI 1.33–3.47 for working more than 52 h; Table 4).

**Table 4:** Logistic regression analysis for dyslipidemia (low-density lipoprotein cholesterol ≥ 130 mg/dl) in the participant children by working hours of the father or mother.

	**Working hours per week**			
	**≤40**	**>40 and ≤52**	**>52**	***P*** **for trend**
**Crude model**				
Father	Ref	1.13 [0.78–1.64]	1.30 [0.90–1.89]	0.169
Mother	Ref	1.48 [0.96–2.29]	1.81 [1.14–2.88]	0.004
Adjusted model^*^				
Father	Ref	0.94 [0.62–1.42]	1.08 [0.71–1.66]	0.733
Mother	Ref	1.58 [0.99–2.51]	2.14 [1.33–3.47]	<0.001

* *Adjusted for age, sex, household income, education level of children and parents, and working hours of father or mother*.

Analyses after stratification by children's school level showed that dyslipidemia in children at the elementary school level was highly associated with mothers' working hours (OR 2.10, 95% CI 1.06–4.16 for working 41–52 h, OR 3.42, 95% CI 1.64–7.14 for working >52 h, *P* for trend <0.001). However, adolescents at the middle or high school level did not show a significant increase in ORs (Table 5).

**Table 5:** Logistic regression analysis for dyslipidemia (low-density lipoprotein cholesterol ≥130 mg/dl) in the participant children by working hours of the father or mother, stratified with educational level of children.

	**Odds ratio (95% confidence interval)**			
	**Working hours per week**		
	**≤40**	**>40 and ≤52**	**>52**	***P*** **for trend**
**Elementary school**				
Father	Ref	1.01 [0.55–1.87]	1.24 [0.63–2.41]	0.550
Mother	Ref	2.10 [1.06–4.16]	3.42 [1.64–7.14]	<0.001
**Middle school**				
Father	Ref	0.55 [0.25–1.19]	0.69 [0.31–1.54]	0.367
Mother	Ref	0.87 [0.33–2.29]	1.76 [0.75–4.09]	0.337
**High school**
Father	Ref	1.12 [0.53–2.38]	1.23 [0.58–2.59]	0.583
Mother	Ref	1.52 [0.71–3.25]	1.80 [0.81–3.98]	0.095

^*^Adjusted for age, sex, household income, education level of parents, and working hours of father or mother.

The same logistic regression analysis adjusting for the same variables in Table 5 with waged workers parents only, dyslipidemia in children showed high ORs with mother's working hours, but did not show statistical significance (OR 1.57, 95% CI 0.74–3.31 for working 41–52 h, OR 2.22, 95% CI 0.85–5.78 for working >52 h, *P* for trend = 0.071). However, after stratification by children's school level, mother's working time >52 h showed the highest association with the children's dyslipidemia (OR 2.91, 95% CI 0.82–10.30 for working 41–52 h, OR 10.90, 95% CI 2.67–44.48 for working >52 h, *P* for trend = 0.001). In the contrast, in the analyses regarding shift work, fathers' night or rotating shift work was associated with dyslipidemia in children (OR 2.81, 95% CI 1.62–4.85, fully adjusted model, Table 6).

**Table 6:** Logistic regression analysis for dyslipidemia (low-density lipoprotein cholesterol ≥130 mg/dl) in the participant children by working shift types of the father or mother.

	**Shift type**
	**Day**	**Fixed evening**	**Night or rotating**
**Number**			
Father	176/2,868 [6.1%]	9/124 [7.3%]	30/300 [10.0%]
Mother	141/2,170 [6.5%]	18/278 [6.5%]	5/79 [6.3%]
**Crude model**					
Father	Ref	1.37 [0.66–2.87]	1.99 [1.22–3.25]
Mother	Ref	1.16 [0.67–2.02]	1.25 [0.40–3.92]
Adjusted model^*^n			
Father	Ref	1.34 [0.56–3.19]	2.81 [1.62–4.85]
Mother	Ref	0.82 [0.45–1.49]	1.07 [0.39–2.96]

* *Adjusted for age, sex, household income, education level of parents, working hours of father or mother, shift types of father or mother*.

## Discussion

In the current study, children of mothers working more than 52 h per week showed 2.14 times higher odds of dyslipidemia compared to 40 h or less, and the association was greater in elementary school students. However, fathers' working time did not show a significant association with their children's LDL-C levels. Unlike fathers, the distribution of mother was not even: the number of the comparison group (2,937 subjects, 77.3%). Therefore, we performed additional analysis in the reference of not working parents. Although the odds ratios from working fathers were higher than mothers in crude models, however, only mothers' group working more than 52 h per week showed high OR (2.34, 95% CI 1.36–4.03) with statistical significance (Supplementary Table 2).

LDL-C is a strong marker for atherosclerosis, and therapeutic decisions in children are based on its level (24). Childhood dyslipidemia, defined as LDL-C ≥ 130 mg/dL, is known to be caused by dietary, genetic, and other secondary factors (25, 26). When more calories than required are consumed, excess calories promote the synthesis of TG and cholesterol in the liver, resulting in an increase in their concentration in the blood. Increased concentration of triglycerides and cholesterol is associated with childhood dyslipidemia, a condition influenced by genetic factors, including polygenic and monogenic defects like familial hypercholesterolemia, familial defective apolipoprotein B or PCSK9, and familial hypertriglyceridemia. Other common causes include obesity, type 2 diabetes mellitus, and nephrotic syndrome.

Since parents play a major role in children's health, their long working hours have been shown to have an effect on the children's health, similar to the findings of this study (27, 28). In particular, among the causes of hyperlipidemia in children, the role of parents is important in monitoring children's diet, and long working hours of parents, which make it difficult to care for children's diet, can show a meaningful association with children's health (29). Especially, this mechanism seems to be more meaningful in that it shows a strong association in the elementary school subgroup in this study.

Mechanisms between parents' working hours and the health impact on children should consider health equity (30). Long working hours can affect workers' health status by reducing time for recovery and rest after work and interfering with healthy behaviors and work-life balance (31). And it also has an association with dietary habits (32). However, since economic difficulties affect the health status to a greater extent, people choose to work for longer hours at the expense of leisure time (33). Similarly, parents' long working hours affect their children's health; thus, mediation through socioeconomic status should also be considered.

The fact that the study results differed depending on whether the father or mother worked for long hours suggests a problematic situation regarding the difference in distribution of duties in the family. The imbalance in the burden of household work between men and women in the family may make women more vulnerable to health risks (34, 35). The results of this study also showed that the effects of long working hours on children's dyslipidemia were associated with a significantly increased risk only for mothers. It is important to note that sex differences in the children's health of long working hours are not simply a woman's problem, but that the imbalance of burden according to gender in the family in Korea can adversely affect children's health.

However, it was noteworthy that rotating shift work of father was associated with dyslipidemia in children. In mothers' results, we observed no association. Possibly fewer number of shift workers in mother could have affected this result. Moreover, reverse association may be considered; for example, mothers who had worked in rotating shift might have quitted or moved to another job because of the burden of childrearing. However, in this study we cannot examine this assumption, further study on this issue is needed.

This study had several strengths. To our knowledge, this is the first study to identify the association between mothers' long working hours and dyslipidemia in their children. In addition, this study used a large sample representative of the general Korean population.

However, this study had several limitations. First, our study is a cross-sectional study and cannot confirm the causal relationship between mothers' long working hours and hyperlipidemia in their children; therefore, further longitudinal studies are needed. Second, since KNHANES cannot include all variables, confounders that were not included in the survey could not be considered in the model in our study. A bias may be taken into account with the recent regulation on 52-h overtime in South Korea. However, as of July 2018, restrictions on 52-h overtime work began for some companies, and the impact on the 2010–2018 data we used is not considered to be important. The indirect measurement of LDL-C levels was a limitation. This value can be influenced by the fasting time and triglyceride concentration. However, considering the structured survey methods of KNHANES and low prevalence of hypertriglyceridemia in children, we believe that this limitation had little effect on the observed results. The potential bias from medical conditions that could cause raise LDL-C level, especially familial hypercholesterolemia should have been considered. The prevalence of familial hypercholesterolemia in Asian countries including Korea is not disclosed currently, but the prevalence is estimated to be around 1/500 (36). Extrapolating this value, 7–8 patients may have been included in the analysis; it is considered that the effect on the overall results was not distorted much by them.

In our study, there were no significant results when father's working hours were set as an exposure variable. When the father's working hours were less than 40 h as the non-exposed group and the more than 52 h as the exposed group, our sample size provided only 14.7% power. Further studies on larger sample sizes are needed.

In conclusion, this study showed an association between mothers' long working hours and children's hyperlipidemia. It was found that there may be a discriminatory effect according to the parent's sex on the health of their children, and the possibility of an imbalance in the burden between men and women in the family was considered. The results of this study emphasize the importance of a working environment that reduces unnecessary long hours by considering not only the health of workers themselves but also the health of their children.

## Data Availability Statement

Publicly available datasets were analyzed in this study. This data can be found here: https://knhanes.kdca.go.kr/knhanes/eng/.

## Ethics Statement

The institutional review board (IRB) of Korea Disease Control and Prevention Agency (KDCA) approved the Korea National Health and Nutrition Examination Survey (KNHANES) with approval numbers 2010-02CON-21-C, 2011-02CON-06-C, 2012-01EXP-01-2C, 2013-07CON-03-4C, and 2013-12EXP-03-5C. Written informed consent to participate in this study was provided by the participants' legal guardian/next of kin. The IRB of Seoul St. Mary's Hospital, the Catholic University of Korea approved with an exemption of ethical revision the design of this study with approval number KC21ZASI0323, because the original data collection was approved by KDCA.

## Author Contributions

MHC and JL designed the study. D-WL, M-YK, and JL performed the statistical analysis. JA and JL wrote the manuscript. J-PM and H-RK interpreted the results and revised the first draft of the manuscript. All authors read and approved the final manuscript before submission. All authors contributed to the article and approved the submitted version.

## Conflict of Interest

The authors declare that the research was conducted in the absence of any commercial or financial relationships that could be construed as a potential conflict of interest.

## Publisher's Note

All claims expressed in this article are solely those of the authors and do not necessarily represent those of their affiliated organizations, or those of the publisher, the editors and the reviewers. Any product that may be evaluated in this article, or claim that may be made by its manufacturer, is not guaranteed or endorsed by the publisher.
