# Supplementary material for: The Association Between Long Working Hours of Parents and Dyslipidemia in Their Children
Source: Front Public Health. 2022 Jun 30;10:894609. doi: 10.3389/fpubh.2022.894609 (PMC9279694; doi:10.3389/fpubh.2022.894609)
Supplement: Supplementary file 1 [file Table_1.DOCX]

**SUPPLEMENTARY TABLE 1.** Distribution of working hours of fathers and mothers of the study participants

|  |  | **Working hours of father per week** | | |  |  |  |  |  |
| --- | --- | --- | --- | --- | --- | --- | --- | --- | --- |
|  |  | **≤ 40** |  | **> 40 and ≤ 52** |  | **> 52** |  | **All** |  |
| **Working hours of** | **≤ 40** | 1,309 | (14.0%) | 221 | (2.4%) | 103 | (1.1%) | 1,633 | (17.5%) |
| **mother per week** | **> 40 and ≤ 52** | 913 | (9.8%) | 139 | (1.5%) | 78 | (0.8%) | 1,130 | (12.1%) |
|  | **> 52** | 715 | (7.6%) | 156 | (1.7%) | 165 | (1.8%) | 1,036 | (11.1%) |
|  | **All** | 2,937 | (31.4%) | 516 | (5.5%) | 346 | (3.7%) | 3,799 | (40.6%) |

**SUPPLEMENTARY TABLE 2.** Logistic regression analysis for dyslipidemia (low-density lipoprotein cholesterol ≥ 130 mg/dL) in the participant children by working hours of the father or mother in reference to not working subjects

|  |  | **Working hours per week** | |  |  |
| --- | --- | --- | --- | --- | --- |
|  |  | **0** | **> 0 and ≤ 40** | **> 40 and ≤ 52** | **> 52** |
| **Number** | **Father** | 18/507  (3.6%) | 71/1,126  (6.3%) | 74/1,130  (6.5%) | 70/1,036  (6.8%) |
|  | **Mother** | 69/1,272  (5.4%) | 94/1,665  (5.6%) | 39/516  (7.6%) | 31/346  (9.1%) |
| **Crude model** | **Father** | Ref | 2.38 [1.29-4.40] | 2.16 [1.17-3.99] | 2.49 [1.35-4.58] |
|  | **Mother** | Ref | 1.16 [0.80-1.67] | 1.61 [0.99-2.63] | 1.98 [1.19-3.29] |
| **Adjusted model*** | **Father** | Ref | 3.15 [0.74-1.67] | 2.86 [0.41-19.90] | 3.29 [0.48-22.69] |
|  | **Mother** | Ref | 1.11 [0.74-1.67] | 1.69 [1.00-2.86] | 2.34 [1.36-4.03] |

*Adjusted for age, sex, household income, education level of parents, and working hours of father or mother
